# Dual-specificity protein phosphatase DUSP4 regulates response to MEK inhibition in *BRAF* wild-type melanoma

**DOI:** 10.1038/s41416-019-0673-5

**Published:** 2019-12-16

**Authors:** Avinash Gupta, Christopher Towers, Frances Willenbrock, Roz Brant, Darren Richard Hodgson, Alan Sharpe, Paul Smith, Anthony Cutts, Anna Schuh, Ruth Asher, Kevin Myers, Sharon Love, Linda Collins, Adelyn Wise, Mark Roy Middleton, Valentine Moya Macaulay

**Affiliations:** 1 Department of Medical Oncology, The Christie NHS Foundation Trust, Manchester, UK; 2 Department of Oncology, Old Road Campus Research Building, University of Oxford, Oxford, UK; 3 Translational Science, Oncology iMED, AstraZeneca, Macclesfield, UK; 4 Oncology iMED, AstraZeneca, Cambridge, UK; 5 Cancer BioSciences, AstraZeneca, Cambridge, UK; 6 Molecular Diagnostics Centre, John Radcliffe Hospital, Oxford University Hospitals NHS Foundation Trust, Oxford, UK; 7 National Institute for Health Research Biomedical Research Centre, Oxford, UK; 8 Department of Cellular Pathology, John Radcliffe Hospital, Oxford University Hospitals NHS Foundation Trust, Oxford, UK; 9 Experimental Cancer Medicine Centre, Oxford, UK; 10 Centre for Statistics in Medicine, Nuffield Department of Orthopaedics, Rheumatology and Musculoskeletal Sciences, University of Oxford, Oxford, UK; 11 Oncology Clinical Trials Office, University of Oxford, Oxford, UK

**Keywords:** Melanoma, Cancer therapeutic resistance, Cell signalling

## Abstract

**Background:**

Aiming to improve treatment options for *BRAF* wild-type melanoma, we previously conducted the DOC-MEK study of docetaxel with MEK inhibitor (MEKi) selumetinib or placebo, revealing trends to prolongation of progression-free survival (hazard ratio 0.75, *P* = 0.130), and improved response rates (32% vs 14%, *P* = 0.059) with docetaxel plus selumetinib. *NRAS* status did not associate with outcome. Here, the aim was to identify novel biomarkers of response to MEKi.

**Methods:**

A MEK 6 gene signature was quantified using NanoString and correlated with clinical outcomes. Two components of the gene signature were investigated by gene silencing in *BRAF/NRAS* wild-type melanoma cells.

**Results:**

In melanomas of patients on the selumetinib but not the placebo arm, two gene signature components, dual-specificity protein phosphatase 4 (DUSP4) and ETS translocation variant 4 (ETV4), were expressed more highly in responders than non-responders. In vitro, ETV4 depletion inhibited cell survival but did not influence sensitivity to MEKi selumetinib or trametinib. In contrast, DUSP4-depleted cells showed enhanced cell survival and increased resistance to both selumetinib and trametinib.

**Conclusions:**

ETV4 and DUSP4 associated with clinical response to docetaxel plus selumetinib. DUSP4 depletion induced MEKi resistance, suggesting that DUSP4 is not only a biomarker but also a mediator of MEKi sensitivity.

**Clinical Trial Registration:**

DOC-MEK (EudraCT no: 2009-018153-23).

## Background

The incidence of melanoma is increasing: it is now the fifth most common cancer in the United Kingdom and the second commonest cancer in adults aged 25–49 (http://www.cancerresearchuk.org/health-professional/cancer-statistics/statistics-by-cancer-type/skin-cancer/). Early detection and treatment with surgical excision is often curative, but ~25% of patients develop local recurrences and/or metastatic disease.^1^ The prognosis with advanced melanoma is poor, although advances in both immunotherapy and targeted therapy have had a significant impact on overall survival (OS), with median OS more than 24 months in some clinical trials.^2^ Approximately 40–50% of melanomas harbour *BRAF* mutations that activate the RAS–RAF–MEK–ERK pathway, also known as the Mitogen-Activated Protein Kinase (MAPK) pathway.^3^ The most common *BRAF* mutation is a missense mutation, leading to substitution of valine by glutamic acid at position 600 of the BRAF protein (V600E).^4,5^ Patients whose melanomas contain these mutations can be treated with BRAF inhibitors, such as vemurafenib or dabrafenib. These drugs achieve ~50% objective response rate (ORR) as monotherapy,^6,7^ and up to 69% ORR when combined with MEK inhibitors (MEKi), which act downstream of BRAF to inhibit the activity of MEK1/2.^8–10^ However, treatment resistance and disease progression generally develop within 6–7 months on BRAF inhibitor (BRAFi) monotherapy and 11 months with BRAFi/MEKi combination treatment. For melanoma patients without *BRAF* mutations, targeted treatments have shown no significant benefit.^11,12^

We previously reported the outcomes of the multicentre Phase II DOC-MEK trial for patients with advanced *BRAF* wild-type melanoma.^13^ Patients were randomised to treatment with docetaxel plus either MEK1/2 inhibitor selumetinib (AZD6244, ARRY-142886) or placebo. The rationales for this combination were, firstly, that selumetinib has demonstrated preclinical efficacy in both *BRAF* wild-type and mutant melanoma models.^14^ Secondly, resistance to taxane-induced apoptosis can be mediated by MAPK pathway activation, and thus concurrent MEK1/2 inhibition may potentiate the efficacy of taxane chemotherapy. Thirdly, the combination of selumetinib and docetaxel has been tested in melanoma and colorectal cancer xenografts^15,16^ and in a Phase I trial,^17^ with evidence of activity in a Phase II study in patients with non-small-cell lung cancer (NSCLC).^18^ In the DOC-MEK trial, patients treated with docetaxel plus selumetinib had an ORR of 32% compared with 14% in the docetaxel plus placebo arm (*P* = 0.059).^13^ There was a non-significant difference in progression-free survival (PFS) and *NRAS* mutation was not predictive of the response to MEK inhibition.^13^

Here, we extend analysis of the DOC-MEK study with reference to a MEK functional activity gene signature, developed as a prognostic and/or predictive biomarker of MEK functional activation across a variety of tumour types.^19,20^ We confirm no evidence of a predictive role for *NRAS* mutation status, but demonstrate that there is a correlation between response to treatment with docetaxel plus selumetinib and the MEK 6 gene signature score. Notably, two components of the signature, Dual-specificity protein phosphatase 4 (DUSP4, also known as MKP2) and ETS translocation variant 4 (ETV4), correlate with partial response (PR) or complete response (CR) to docetaxel plus selumetinib but not to docetaxel plus placebo. This suggests that these proteins could act as biomarkers to identify patients likely to respond to the combination treatment. We investigate a possible role for DUSP4 and ETV4 in mediating this response by assessing the effect of their depletion on the sensitivity of *BRAF* wild-type melanoma cells to MEK inhibition. We show that DUSP4 protein expression is suppressed by MEK inhibition, confirming its status as an ERK-regulated gene.^21^ Furthermore, we demonstrate that DUSP4 depletion influences response to two MEK inhibitors, selumetinib and trametinib. Thus, DUSP4 expression is not only a potential biomarker for patient response to MEK inhibition but also a mediator of MEK inhibitor sensitivity.

## Methods

### Tumour mutation analysis by next-generation sequencing

Archival formalin-fixed paraffin-embedded (FFPE) tissue blocks were sectioned and enriched for tumour tissue by macro-dissection. Genomic DNA was extracted by using the QIAamp FFPE Tissue kit and amplified using the Ion Ampliseq^TM^ Library Kit 2.0. DNA sequencing was performed by the IonTorrent Personal Genome Machine (LifeTechnologies, Carlsbad, CA). A targeted cancer hotspot panel (designed using the Ion Ampliseq^TM^ Cancer Primer Pool) was used to detect mutations in 46 known cancer-related genes (Supplementary Table 1).^22^ The sensitivity of this assay is 5–10% (% of mutant DNA detectable in a background of wild-type DNA). When DNA was of insufficient quality for next-generation sequencing (NGS), pyrosequencing was used to test for mutations in codons 12, 13 and 61 of *NRAS*.

### Gene expression analysis

Tumour FFPE tissue was macro-dissected from 1 to 2 × 5-µm sections, RNA extracted using the RNeasy FFPE kit according to the manufacturer’s instructions and 100 ng of each RNA was analysed using the NanoString nCounter gene expression system.^23^ The code set was designed by NanoString Inc. (Seattle, WA). Transcript counts were normalised between MEK signature genes and reference genes and transformed using the NanoString Normalisation Tool v2 (AstraZeneca Oncology Bioinformatics http://CRAN.R-project.org/package=NAPPA) in order to generate signature scores.^20^ Signature scores were calculated blind to clinical outcomes.

### Cell lines and reagents

CHL-1 cells were obtained from the American Type Culture Collection (ATCC), and SK-mel-23 cells from Professor V. Cerundolo, Weatherall Institute of Molecular Medicine, University of Oxford. Cultures were maintained in Dulbecco’s Modified Eagle’s Medium with 10% foetal calf serum and 1% penicillin/streptomycin, in a humidified atmosphere of 10% CO_2_. Both cell lines were negative for mycoplasma (MycoAlert kit, Lonza Rockland Inc., Rockland, USA), and were authenticated by STR genotyping (Eurofins Medigenomix Forensik GmbH). Selumetinib and trametinib (Selleck) were stored as 10 mM solutions in DMSO at −80 °C.

### Western blotting and cell survival assays

Cells were incubated with drugs for 60–150 min before harvesting for western blotting as previously described,^24^ using antibodies to DUSP4 (♯5149, Cell Signaling Technology (CST)), phospho-T202/Y204 ERK 1/2 (♯4377, CST), total ERK 1/2 (♯4695, CST), β-tubulin (T4026, Sigma) and actin (A3854, Sigma). For clonogenic survival assays, drugs or vehicle control were added 24 h after seeding, and cells incubated in the presence of drug for 7–14 days.

### Gene silencing by siRNA transfection

Cells were reverse transfected with 50 nM gene-specific or non-silencing Allstars (Qiagen) siRNA on day 1 using Dharmafect 1 (ThermoFisher), forward transfected with 50 nM siRNA on day 2 and re-seeded on day 3 for clonogenic assays. DUSP4 was depleted using siDUSP4_1 (♯4392420-s4372, Ambion) and siDUSP4_2 (J-003963-09, Dharmacon) and ETV4 using siETV4_1 and _2 (♯106636 and ♯106637, Thermofisher).

### Quantitative real-time PCR (qRT-PCR)

RNAs were extracted using the Reliaprep^TM^ RNA miniprep kit (Promega) and reverse-transcribed to complementary DNA (cDNA) using Superscript III First-Strand Synthesis Supermix (ThermoFisher). PCRs were performed on a 7500 Fast RT-PCR System (Applied Biosystems) using SYBR Green PCR mastermix (ThermoFisher) with the following primers: DUSP4 forward, 5′-GGGGTCCTGTGGAGATCCTT-3′ and reverse, 5′-GGCAGTCCGAGGAGACATTC-3′; ETV4 forward 5′-GAGCGGAGGATGAAAGCCG-3′ and reverse 5′-CCCATTTCCGGGCGATTTG-3′; TUBA6 (housekeeping gene) forward 5′-CCCCTTCAAGTTCTAGTCATGC-3′ and reverse 5′-ATTGCCAATCTGGACACCA-3′.

### Statistical analysis

The Kaplan–Meier method was used to obtain PFS and OS estimates by mutation status. As reported previously, the impact of *NRAS* mutation status was assessed by adding an interaction term with treatment in the Cox model, adjusting for stratification variables and using a significance level of 0.05.^13^ Correlations between tumour mutation results, gene signature scores and clinical outcomes were analysed using Microsoft Excel, with significance determined using *t* tests with a one-tailed distribution. *NRAS* VAF was compared with gene signature scores using Pearson’s correlation coefficient. Western blot and cell survival data were analysed using GraphPad Prism v5, using *t* tests to compare two groups and ANOVA for multiple groups in each case with a two-tailed distribution.

## Results

### Response to MEK inhibition does not correlate with NRAS mutation status

**Fig. 1:**
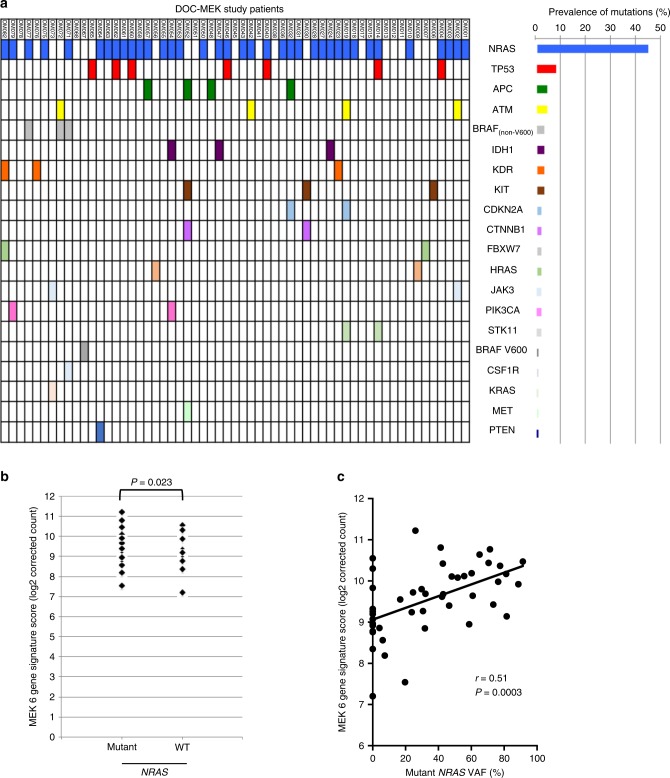
Correlation of *NRAS* mutations with MEK 6 gene signature score. **a** Distribution of mutations detected in samples from 59 patients using the 46-gene cancer panel. **b** MEK 6 gene signature scores of patient samples with mutant or wild-type *NRAS*. **c** Correlation between MEK 6 gene signature score and variant allele frequency of *NRAS*.

The results from the DOC-MEK Phase II trial in *BRAF* wild-type advanced melanoma patients^13^ demonstrated that docetaxel plus selumetinib did not significantly increase PFS or OS compared with docetaxel plus placebo, but there was a trend towards increased ORR in the selumetinib group (*P* = 0.059). There was no correlation between patient response and *NRAS* mutation status. Here, we extended this analysis by investigating the mutational status of 45 additional genes in a 46-gene cancer panel. Mutation status was determined for 64 of the 83 patients randomised in the DOC-MEK study, in 59 cases by NGS using targeted sequencing of 46 cancer-associated genes (Fig. 1a, Supplementary Table 1) and in five cases (DM005, DM026, DM029, DM037 and DM083) using pyrosequencing. In total, 84 mutations were found in 49 of the 59 cases analysed using NGS, with two or more concurrent mutations found in 24 cases. The most commonly mutated gene was *NRAS* (45% of all mutations detected), followed by *TP53* (7%; Fig. 1a, Supplementary Table 2). These findings are consistent with an analysis of 699 unselected (i.e. *BRAF* wild-type and mutated) melanomas using the same 46-gene cancer panel, where, after *BRAF* mutations (41% of cases), *NRAS* (22%) and *TP53* (17%) were the next commonest mutations.^22^ In our *BRAF* wild-type population, four cases tested by NGS had a *BRAF* mutation that had not been detected during initial screening by hotspot mutation testing (Fig. 1a, Supplementary Table 2). One of these was *BRAF* V600E, detected at a very low variant allele frequency (VAF) of 5.5%. Three cases harboured non-V600 *BRAF* mutations: K601E, G466R and N581S, rare mutations reported in the COSMIC database (http://cancer.sanger.ac.uk). Non-V600 *BRAF* mutations, including K601E, have been associated with sensitivity to MEK inhibition.^25,26^ The G466R and N581S *BRAF* mutations were found in melanomas that also harboured mutant *NRAS*. As activating *BRAF* and *NRAS* mutations are considered mutually exclusive,^4^ these two *BRAF* mutations are probably non-activating, or may be low-activity *BRAF* mutants that require upstream RAS activation. The patient with low VAF *BRAF* V600E (case DM067, Supplementary Table 2) was randomised to docetaxel plus placebo and progressed within 3.3 months of treatment. Those with non-V600 *BRAF* mutations were all randomised to docetaxel plus selumetinib. The patients with *BRAF* K601E (case DM072) and BRAF G466R + *NRAS* Q61H mutant melanoma (case DM071) progressed after 4 and 7 months, respectively, each with stable disease as best response. The patient with *BRAF* N581S melanoma (case DM077) was found to have two concurrent *NRAS* mutations (Q61K, Q61R) and initially demonstrated a partial response to treatment, but again progressed quickly after 4 months. The most common concomitant mutations were *NRAS* and *TP53* (Supplementary Table 2). There was no apparent association between the number of concomitant mutations per tumour and median PFS or OS (Supplementary Table 3).

### Relationship between NRAS mutation status and MEK 6 gene expression signature

Forty-eight tumours from the DOC-MEK study were available for transcriptional analysis, using the NanoString platform to quantify the MEK 6 gene score. Supplementary Table 4 summarises these scores, the *NRAS* mutation data and DOC-MEK clinical outcomes. We tested for associations between the MEK 6 gene score and *NRAS* status, best overall response and derived benefit. There was a higher mean MEK 6 gene score in *NRAS* mutant melanomas compared with *NRAS* wild-type melanomas (*P* = 0.023, Fig. 1b), but the differences were small and there was considerable overlap between the two groups. Since the levels of MAPK activation may be significantly different between tumours with low vs high mutant *NRAS* VAF, we assessed the correlation between *NRAS* VAF where available and MEK 6 gene score and found a modest positive correlation (Pearson’s correlation coefficient, *r* = 0.51, Fig. 1c).

### Response to MEK inhibition correlates with MEK 6 gene expression score

**Fig. 2:**
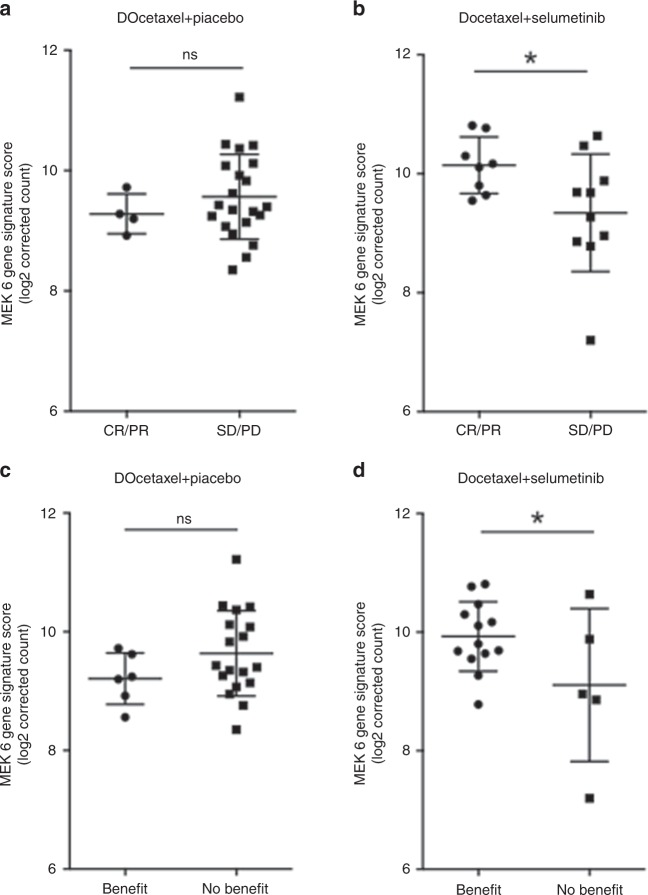
Correlation of response to docetaxel plus selumetinib with MEK 6 gene signature score. **a** Best overall response for patients treated with docetaxel + placebo comparing CR and PR with SD and PD. **b** Best overall response for patients treated with docetaxel + selumetinib as in (**a**), *p = 0.026 using one-tailed *t* test. **c** Derived benefit for patients treated with docetaxel + placebo comparing benefit with no benefit. **d** Derived benefit for patients treated with docetaxel + selumetinib as in **c**, *p = 0.038 using one-tailed *t* test, testing the pre-established hypothesis that a higher MEK 6 gene score correlates with better clinical outcome when treated with a MEK inhibitor.

To assess correlations between patient response and MEK 6 gene expression score, patients in the two arms of the trial were analysed separately by comparing those achieving CR/PR with patients having stable disease (SD) or progressive disease (PD) at first assessment (Fig. 2a, b). For patients treated with docetaxel plus placebo, there was no significant difference in MEK 6 gene score between responders and those with SD/PD at first assessment (mean scores 9.28 ± 0.16 and 9.56 ± 0.15, respectively, *P* = 0.222, Fig. 2a). In contrast, patients achieving CR or PR on docetaxel plus selumetinib had a higher MEK 6 gene score than those with SD/PD at first assessment (mean scores 10.14 ± 0.17 and 9.34 ± 0.31, respectively, *P* = 0.026, Fig. 2b). However, there was again considerable overlap between the two populations. The absolute difference between mean scores was small, and as there were only four responding patients in the docetaxel plus placebo arm, these results should be interpreted with caution.

Since patients with prolonged disease stabilisation can also be considered to have derived benefit from treatment, a second analysis was performed. Gene expression scores were compared between those who “derived benefit”, i.e. had CR, PR or SD ≥ 6 months, and those with “no derived benefit”, i.e. PD at first assessment or SD < 6 months (Fig. 2c, d). As previously, for patients treated with docetaxel plus placebo, there was no difference in the MEK 6 gene score between those who derived benefit and those who did not (mean values of 9.21 ± 0.18 and 9.64 ± 0.17, respectively, *P* = 0.094, Fig. 2c). With more patients in the “benefit” group, this analysis may be considered more reliable. Patients treated with docetaxel plus selumetinib who were considered to have derived benefit again had a higher MEK 6 gene score than those who did not benefit (mean scores 9.93 ± 0.16 and 9.11 ± 0.58, respectively, *P* = 0.038, Fig. 2d), with a similar pattern of distribution as in Fig. 2b. Thus, using either approach to categorise clinical benefit, this analysis showed a higher mean MEK 6 gene score in the melanomas of patients responding to docetaxel plus selumetinib, but not to docetaxel plus placebo. However, the absolute difference in mean score using either approach was small.

In a third approach, we analysed expression of individual components of the MEK 6 gene signature, comparing those who responded (CR plus PR) with those with PD at first assessment. Gene expression data were available for 8/13 responders and 2/5 experiencing early progression in the combination treatment arm, and for 3/6 responders and 11/20 experiencing early PD in the docetaxel plus placebo arm. Whilst the numbers are small, the MEK 6 gene score data revealed potentially interesting differences in the expression of DUSP4 and ETV4, which showed significant differences in mean values (*p* < 0.05) between the responders and nonresponders in the selumetinib group (Table 1), but not in the placebo group (Supplementary Table 5). Thus, patients who responded to treatment had greater expression of both DUSP4 and ETV4 compared with those experiencing early PD, and these differences were found only in the docetaxel plus selumetinib group.

**Table 1:** Expression of individual genes in the MEK 6 gene score in melanomas of patients treated with docetaxel plus selumetinib.

Grey shaded rows: patients with PR/CR to docetaxel plus selumetinib combination treatment; unshaded rows: patients with PD at first assessment.

Mean gene expression in both groups was compared using Student’s *t* test with two-tailed distribution.

^a^Cases where ETV4 expression was below the set limit of detection (mean minus two standard deviations), so accuracy is unclear. Bold values are the mean figures for the gene expression values per gene in each group.

Since higher expression of DUSP4 and ETV4 mRNA was associated with clinical response to MEK inhibition, we hypothesised that depletion of each of these proteins might have the reverse effect and induce resistance to MEK inhibition. We therefore decided to test this in vitro using *BRAF* wild-type melanoma cell lines CHL-1 and SK-MEL-23, and two MEK inhibitors, namely selumetinib, the MEK inhibitor used in the DOC-MEK clinical trial, and trametinib, the first MEK inhibitor approved by the FDA for use in clinical practice (https://www.cancer.gov/about-cancer/treatment/drugs/fda-trametinib). The aim of using two inhibitors was to check whether any observed changes were likely to be class effects, rather than specific to selumetinib. Western blots for phospho-T202/Y204 ERK 1/2 levels were used as a readout for MEK activity.

### MEK inhibition downregulates DUSP4 in BRAF wild-type melanoma cells

**Fig. 3:**
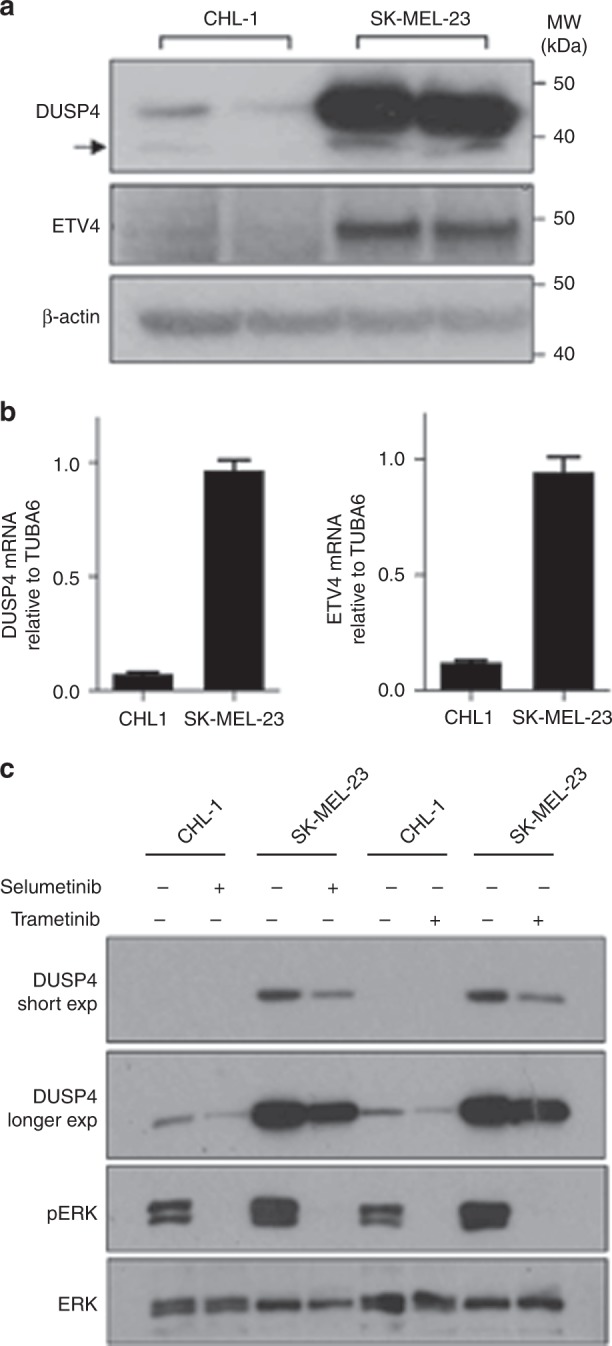
MEK inhibition decreases ERK phosphorylation and DUSP4 expression in *BRAF* wild-type melanoma cell lines. **a** Endogenous expression of DUSP4 and ETV4 protein in duplicate whole-cell extracts of CHL-1 and SK-MEL-23 cells analysed by western blotting. Similar results were obtained in *n* = 3 independently prepared lysates. A faint ~40-kDa band (arrowhead) just below DUSP4 band was not reduced by DUSP4 depletion (see Fig. 4b, c) so it may be non-specific. **b** *DUSP4* and *ETV4* mRNA quantified by qRT-PCR (*n* = 3 independently prepared cDNAs). **c** CHL-1 and SK-MEL-23 cells were treated with 100 nM selumetinib or 10 nM trametinib for 1 h before analysis by western blot. Supplementary Fig. 1A–C shows concentration and time dependence of response to MEK inhibition.

We first assessed endogenous expression of DUSP4 and ETV4, which were detectable at the protein and mRNA level in both cell lines (Fig. 3a, b), although CHL-1 showed considerably lower levels of expression of both proteins than SK-MEL-23. We also tested the effect of MEK inhibition on DUSP4 expression, using both selumetinib and trametinib. Selumetinib inhibited ERK phosphorylation at ≥100 nM, whereas trametinib caused inhibition at lower concentrations (≥3 nM) in both cell lines (Fig. 3c, Supplementary Fig. 1A, B), in keeping with the known IC_50_ values for each drug.^14,27^ In both cell lines, DUSP4 expression also decreased as inhibitor concentration increased (Fig. 3c, Supplementary Fig. 1A, B), consistent with reported control of its transcription by phosphorylated ERK1.^21^ Trametinib suppressed ERK phosphorylation for 24 h, with partial return of signal at 48–72 h, while inhibition of DUSP4 expression persisted for at least 72 h (Supplementary Fig. 1C).

### ETV4 depletion does not alter sensitivity of BRAF wild-type melanoma cells to MEK inhibition

We assessed the influence of ETV4 on response to MEK inhibition, by depleting ETV4 and measuring cell survival and MEK inhibitor SF_50_ values (drug concentration suppressing survival to 50% of control values) in clonogenic survival assays. ETV4 knockdown was very effective in CHL-1 cells, with residual ETV4 mRNA values of 1.16 ± 0.53% and 4.14 ± 0.65% for siETV4_1 and siETV4_2, respectively, compared with the Allstars control siRNA transfectants (Supplementary Fig. 2A, left). For SK-MEL-23 cells, the equivalent values were 18.02 ± 13.02% and 4.28 ± 1.84% (Supplementary Fig. 2A, right). ETV4 depletion had a largely detrimental effect on cell survival in SK-MEL-23 but not in CHL-1 cells (Supplementary Fig. 2B). Supplementary Fig. 2C, D shows the results of representative survival assays testing the effects of ETV4 depletion on response to trametinib, and summarises SF_50_ values from three independent assays in each cell line. We found no evidence that ETV4 depletion influenced the response of either cell line to MEK inhibition by trametinib (Supplementary Fig. 2C, D). However, the presence of relatively few surviving colonies in ETV4-depleted cultures, especially of SK-MEL-23 (Supplementary Fig. 2B, right), could have contributed to the variation in trametinib SF_50_ data (Supplementary Fig. 2D), so we cannot exclude a small effect on trametinib response.

### DUSP4 depletion induces resistance of BRAF wild-type melanoma cells to MEK inhibition

**Fig. 4:**
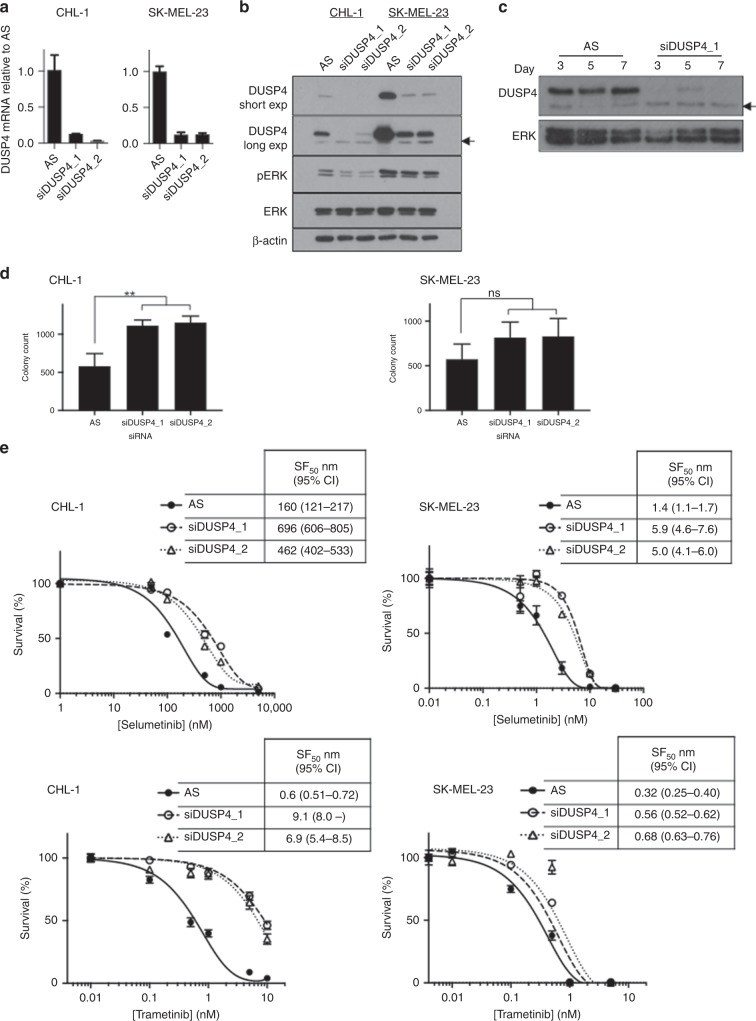
Depletion of DUSP4 decreases sensitivity to MEK inhibition in *BRAF* wild-type melanoma cell lines. **a**, **b** Quantification of DUSP4 depletion following knockdown by siDUSP4_1 and siDUSP4_2 in: left, CHL-1 and right, SK-MEL-23 cells analysed by qRT-PCR (**a**) and western blot (**b**). Arrowhead: probable non-specific band not reduced by DUSP4 depletion. **c** Duration of DUSP4 knockdown in CHL-1 cells, analysed by western blotting at 3, 5 and 7 days after transfection. Representative results are shown for Allstars (AS) control siRNA and siDUSP4_1. ERK is shown as a loading control. **d** The effect of DUSP4 depletion by siDUSP4_1 and siDUSP4_2 on colony count in: left, CHL-1 and right, SK-MEL-23 cells (mean ± SEM of five independent experiments). **e** The effect of DUSP4 depletion by siDUSP4_1 and siDUSP4_2 on sensitivity of: left, CHL-1 and right, SK-MEL-23 cells to MEK inhibitors selumetinib and trametinib (mean ± SEM of triplicate values for a single representative experiment). Table 2 shows a summary of fold changes in MEK inhibitor sensitivity (pooled data from six experiments in each case).

Next, we used siRNAs to deplete DUSP4 and measured cell survival and MEK inhibitor SF_50_ values. Both DUSP4 siRNAs used induced effective DUSP4 depletion, at both the mRNA and protein level (Fig. 4a, b). We tested the duration of DUSP4 knockdown in CHL-1 cells and demonstrated that depletion lasted at least 7 days (Fig. 4c). Compared with controls, DUSP4-depleted cells showed an increase in cell survival that was significant in CHL-1 cells (**P = 0.0187 and 0.0154 for siDUSP4_1 and _2, respectively, Fig. 4d left), but not in SK-MEL-23 cells (Fig. 4d right).

Finally, we tested the sensitivity of DUSP4-depleted cells to both MEK inhibitors. The results are shown in Fig. 4e, and the data are summarised in Table 2. In both cell lines with both MEK inhibitors, there was a consistent shift to the right of the MEK inhibitor dose–response curve (Fig. 4e), with 2.5–6.8-fold increase in SF_50_ values in DUSP4-depleted cells compared with controls (Table 2). These results indicate that DUSP4-depleted cells were less sensitive to both selumetinib and trametinib than the control transfectants. Thus, DUSP4 protein levels are not only affected by MEK inhibition but also alter cellular response to this class of drug.

**Table 2:** Effect of DUSP4 depletion on response to MEK inhibition in BRAF wild-type melanoma cells.

Cell line	CHL-1		SK-MEL-23	
MEK inhibitor	Selumetinib	Trametinib	Selumetinib	Trametinib
Fold increase in SF50	6.71 ± 1.30	6.85 ± 2.42	2.94 ± 0.40	2.48 ± 0.44
*p*-value	0.0072	0.0605	0.0048	0.02

The table shows mean ± SEM fold increase in SF_50_ values in DUSP4-depleted CHL-1 and SK-MEL-23 cells (*n* = 6 in each case; data analysed using two-tailed *t* test).

## Discussion

There are fewer options for treating *BRAF* wild-type advanced melanoma than for the *BRAF*-mutated population. DOC-MEK was the first published randomised trial in a selected *BRAF* wild-type melanoma population, and indicated that a proportion of such patients could benefit from combined treatment with docetaxel and selumetinib.^13^ During the conduct of the DOC-MEK study, MEK inhibitor binimetinib (MEK162) was reported to have a 20% ORR (6/30) in patients with *NRAS*-mutated melanoma in a monotherapy Phase II study.^28^ However, in a retrospective analysis of DOC-MEK data, we observed no correlation between *NRAS* status and clinical outcome in either treatment arm,^13^ reflecting previously published data with selumetinib alone^29^ and selumetinib plus dacarbazine/docetaxel chemotherapy.^30^

We extended the mutational analysis to a 46-gene cancer panel and found no correlation between the number of concomitant mutations and clinical outcome. We therefore investigated the hypothesis that the expression of genes that correlate with increased MAPK pathway activity may predict for sensitivity to MEK inhibitor therapy. Whilst *BRAF* and *RAS* mutations vary across cell lines that are sensitive to MEK inhibition, a MEK 18-gene functional activation signature score was previously found to be consistently elevated in selumetinib-sensitive cell lines, and was higher in *BRAF* mutant vs wild-type melanomas.^19^ These 18 genes are *DUSP4, DUSP6, ETV4, ETV5, PHLDA1, SPRY2, ELF1, FXYD5, KANK1, LGALS3, LZTS1, MAP2K3, PROS1, S100A6, SERPINB1, SLCO4A1, TRIB2* and *ZFP106*. After refining the score to six genes (*DUSP4, DUSP6, ETV4, ETV5, PHLDA1,* and *SPRY2*), based on reproducibility across tumour types, the MEK 6 gene score has been shown to be higher in *KRAS* mutant than *KRAS* wild-type NSCLC.^20^ This suggests that known activating mutations in the MAPK pathway are associated with higher MEK gene signature scores. Indeed, all the components of the 6-gene score are known transcriptional targets of the MEK–ERK pathway.^21,31–36^ Furthermore, four of six of these genes (*DUSP4, DUSP6, PHLDA1* and *SPRY2*) are negative regulators of ERK pathway activity, forming part of a regulatory feedback loop.^21,31,33,36,37^ Consistent with the ability of mutant *NRAS* to activate MEK–ERK signalling, our results suggest that *NRAS* mutant melanoma is associated with a higher MEK 6 gene expression score than *NRAS* wild-type melanoma (Fig. 1b). It is interesting to note that the second highest MEK 6 gene score was in a melanoma found to have two concurrent *NRAS* mutations, Q61K and Q61R (Supplementary Table 4, patient DM077). However, neither *NRAS* mutation status nor VAF analysis was sufficiently discriminating to judge dependence on the MAPK pathway and thus potential sensitivity to MEK inhibition. We then assessed the MEK score with respect to patient outcome, and here the data suggested that a higher MEK score did predict for sensitivity to selumetinib plus docetaxel combination therapy, but not docetaxel therapy alone (Fig. 2). To strengthen this conclusion, it would have been preferable to obtain on-treatment biopsies, to confirm that MEK–ERK was indeed inhibited by selumetinib. We also acknowledge that there were only minor differences in MEK signature score by *NRAS* mutation and clinical response status, limiting the utility of this score as a biomarker for MEK inhibitor response in melanoma. Further data would be required to confirm this trend and fully characterise an optimal threshold.

We report here that two components of the MEK 6 gene score, ETV4 and DUSP4, were expressed at significantly higher levels in melanomas of responders to docetaxel plus selumetinib compared with those who progressed at first assessment. This difference was not found in the docetaxel plus placebo arm. This suggests the possibility that DUSP4 and ETV4 may be potential biomarkers of sensitivity to MEK inhibition. We wished to extend this observation by ascertaining whether DUSP4 or ETV4 might also influence the response of wild-type *BRAF* melanoma cells to MEK inhibition. ETV4 is a member of the polyomavirus enhancer activator 3 (PEA3) subfamily of the Ets transcription factor family and regulates genes that promote metastasis.^38^ As well as inducing ETV4 expression, ERK1/2 promotes ETV4 activation by phosphorylation and sumoylation.^34,39,40^ Previous studies reported inconsistent findings regarding the contribution of ETV4 to cell survival.^41,42^ Our data indicate that ETV4 depletion inhibited cell survival of *BRAF* wild-type melanoma cells but did not influence response to MEK inhibition.

DUSP4 was the only other component of the MEK 6 gene score that was expressed at significantly higher levels in the melanomas of patients who responded to selumetinib and docetaxel, compared with those who progressed at first assessment (Table 1). DUSP4 dephosphorylates and thus inactivates ERK1/2 in the nucleus, and may also act on the JNK and p38 pathways.^43^ There is conflicting evidence regarding the significance of DUSP4 expression in cancer. DUSP4 upregulation has been reported in *KRAS* mutant rectal cancer,^44^ and higher DUSP4 levels have been found in melanoma cell lines compared with normal human epidermal melanocytes.^45^ Conversely, DUSP4 levels are higher in indolent ovarian serous borderline tumours compared with more aggressive serous carcinomas,^46^ and silencing of DUSP4 plays a key role in the development of glioblastomas,^47^ suggesting a tumour-suppressor role. In vitro, DUSP4 knockdown increases growth of EGFR mutant lung adenocarcinoma cell lines, whereas in colorectal cancer cell lines DUSP4 overexpression results in increased proliferation.^48^ There is also conflict in the literature regarding the significance of DUSP4 for predicting response to anticancer therapy. Higher DUSP4 expression has been found to correlate with resistance to anti-EGFR antibody cetuximab in patients with metastatic colorectal cancer, although this may simply reflect the presence of *KRAS* mutations that activate RAS–MAPK.^49^ Conversely, lower DUSP4 expression in breast cancer was reported to be associated with reduced response to neoadjuvant chemotherapy, and in breast cancer cell lines, DUSP4 depletion increased resistance to docetaxel and other cytotoxic drugs, while overexpression increased chemotherapy-induced apoptosis.^50^

Our data are consistent with findings that endogenous DUSP4 levels vary between melanoma cell lines in vitro.^51^ Relative overexpression of DUSP4 and ETV4 in SK-MEL-23 compared with CHL-1 may reflect the fact that SK-MEL-23 cells harbour amplified wild-type *BRAF.*^52^ Both cell lines used in this study harbour wild-type *NRAS*; given that ~60% of patients in the clinical DOC-MEK study had *NRAS* mutant melanoma, it may be informative to assess the contribution of DUSP4 and ETV4 to MEK inhibitor response in *NRAS* mutant cell lines. In the Broad-Novartis Cancer Cell Line Encyclopedia (CCLE) database, melanoma cell lines express the highest level of DUSP4 mRNA of all cancer cell lines tested, likely reflecting the importance of RAS–RAF–MEK activation in melanomas (www.broadinstitute.org/ccle). Of relevance to our study, CCLE data indicate that high DUSP4 expression is predictive of increased sensitivity to selumetinib, with an odds ratio of 2.3.^53^ Similarly, in a panel of pan-negative (wild-type BRAF, NRAS, KIT, GNAQ and GNA11) melanoma cell lines, cells expressing higher levels of DUSP4 have been reported to show significantly greater sensitivity to MEK inhibition.^51^ This correlates with our finding that CHL-1 cells that express lower DUSP4 were less sensitive to selumetinib than the higher-expressing SK-MEL-23 cell line (Fig. 4e). We further found that DUSP4 expression was reduced by MEK inhibition, confirming that DUSP4 levels are regulated by the MAPK pathway.^21^ Consistent with this, and given that DUSP4 is a negative regulator of the MAPK pathway, depleting DUSP4 increased survival of CHL-1 cells. DUSP4 depletion did not influence survival of SK-MEL-23 cells, possibly because residual DUSP4 may have been sufficient to maintain MAPK pathway regulation, consistent with data in Fig. 4b.

Finally, we showed that siRNA-mediated DUSP4 depletion leads to desensitisation to MEK1/2 inhibition. This response parallels the finding in the clinical trial, where higher DUSP4 expression associated significantly with response to MEK inhibition. We observed the MEK sensitisation effect in CHL-1 cells, where DUSP4 depletion had influenced cell survival, and also in SK-MEL-23 cells, where DUSP4-depleted cells showed no significant difference in cell survival compared with controls. These results suggest that the effect of DUSP4 on MEK inhibitor sensitivity is likely independent of the effect on cell survival. Thus, while previous studies showed a correlation between DUSP4 expression and MEK inhibitor sensitivity in pan-tumour cell line panels,^53^ we report for the first time that this association exists in clinical melanomas, and depleting DUSP4 expression induces resistance to MEK inhibition. These results suggest that DUSP4 is capable of influencing response to drugs that target MEK. Furthermore, given that BRAF amplification reportedly mediates MEK inhibitor resistance^54^ the ability of DUSP4 depletion to sensitise SK-MEL-23 cells suggests that this approach may have merit in the BRAF-amplified population. However, we recognise the need to be cautious in interpretation of our data, given the small size of the clinical study and in vitro analysis. It may be informative to assess the predictive significance of DUSP4 in a larger clinical dataset.

In summary, our findings suggest that DUSP4 plays a direct role in determining cellular response to MEK inhibition. DUSP4 may therefore be not only a biomarker for, but also a potential determinant of, the response of wild-type *BRAF* melanomas to MEK inhibition.

## Supplementary information

Supplementary material

## Data Availability

The dataset used and analysed for this paper is available from the corresponding author on request.
