# Supplementary material for: Dual-specificity protein phosphatase DUSP4 regulates response to MEK inhibition in BRAF wild-type melanoma
Source: Br J Cancer. 2019 Dec 16;122(4):506–16. doi: 10.1038/s41416-019-0673-5 (PMC7028919; doi:10.1038/s41416-019-0673-5)
Supplement: Supplementary file 1 — Supplementary material [file 41416_2019_673_MOESM1_ESM.docx]

**Dual specificity protein phosphatase DUSP4 regulates response to MEK inhibition in *BRAF* wild-type melanoma**

A. Gupta, C. Towers, F. Willenbrock, R. Brant, D.R. Hodgson, A. Sharpe, P.D. Smith, A. Cutts, A. Schuh, R. Asher, K. Myers, S. Love, L. Collins, A. Wise, M.R. Middleton, V.M. Macaulay

**Supplementary Data**

SupplementaryTables

| Genes validated for diagnostic use | Genes presented on a research basis | | | |
| --- | --- | --- | --- | --- |
| BRAF | ABL1 | ERBB2 | HRAS | NPM1 |
| EGFR | AKT1 | ERBB4 | IDH1 | PTPN11 |
| KIT | ALK | FBXW7 | JAK2 | RB1 |
| KRAS | APC | FGFR1 | JAK3 | RET |
| NRAS | ATM | FGFR2 | KDR | SMAD4 |
| PDGFRA | CDH1 | FGFR3 | MET | SMARCB1 |
| PIK3CA | CDKN2A | FLT3 | MLH1 | SMO |
| PTEN | CSF1R | GNAS | MPL | SRC |
| TP53 | CTNNB1 | HNF1A | NOTCH1 | STK11 |
|  |  |  |  | VHL |

**Supplementary Table 1:** **Genes in 46 cancer gene panel.** Hotspot mutation detection was performed using IonTorrent Personal Genome Machine. Assays for mutations in nine of these genes have been validated for clinical use and the remaining assays are available for research use.

**Supplementary Table 2:** **Number and type of mutations detected per case:** Distribution of mutations detected in samples from 59 patients tested using the 46 gene cancer panel.

| **No. of mutations** | **0** | **1** | **≥ 2** |
| --- | --- | --- | --- |
| **No. of cases** | 9 | 24 | 24 |
| **Median PFS (months, PP population)** | 4.27 | 4.39 | 4.14 |
| **Median OS (months, PP population)** | 10.43 | 11.65 | 9.30 |

**Supplementary Table 3: Summary of mutations detected per sample and patient outcome.** PFS, progression free survival; OS, overall survival; PP, per protocol. There were no significant differences in PFS or OS between patients whose melanomas contained 0, 1 or ≥2 mutations (p>0.05 by one-way ANOVA).

**Supplementary Table 4: Summary of gene expression data correlated with clinical outcome.** *NRAS* status was determined by NGS using the 46 gene cancer panel. WT, wild-type; PD, progressive disease; SD, stable disease; PR, partial response; CR, complete response; NA, not available; c, censored.

| **Trial no.** | **Normalised values** | | | | | |
| --- | --- | --- | --- | --- | --- | --- |
|  | **DUSP4** | **DUSP6** | **ETV4** | **ETV5** | **PHLDA1** | **SPRY2** |
| **PR/CR** | | | | | | |
| **DM028** | 9.887194 | 10.955445 | 8.085209 | 8.990286 | 10.997901 | 9.386733 |
| **DM030** | 8.934151 | 8.182466 | 8.340894 | 10.365277 | 10.358002 | 7.321323 |
| **DM052** | 10.009136 | 7.743555 | 7.967867 | 11.215295 | 9.771230 | 8.513439 |
| **Mean** | **9.610160** | **8.960489** | **8.131323** | **10.190286** | **10.375711** | **8.407165** |
| **PD** | | | | | | |
| **DM007** | 9.495310 | 10.402783 | 9.047305 | 9.785372 | 11.260327 | 8.960709 |
| **DM019** | 10.720741 | 10.895120 | 8.515907 | 9.836377 | 10.748692 | 9.988248 |
| **DM028** | 8.939076 | 9.317774 | 7.439848 | 9.508937 | 11.026119 | 8.633325 |
| **DM029** | 8.873473 | 10.139463 | 7.626767 | 10.602202 | 11.011783 | 7.857903 |
| **DM041** | 10.544330 | 7.109423 | 7.730938 | 10.536587 | 10.183001 | 8.332260 |
| **DM043** | 10.937361 | 11.041192 | 9.230746 | 10.540165 | 11.073405 | 9.404785 |
| **DM048** | 6.692641 | 9.119352 | 9.004512 | 9.591143 | 9.186803 | 8.991086 |
| **DM051** | 8.481253 | 9.441539 | 9.363928 | 10.993551 | 10.513146 | 7.128626 |
| **DM054** | 10.112275 | 10.847430 | 6.951539 | 11.284806 | 10.981491 | 10.297288 |
| **DM060** | 10.225808 | 8.197017 | 6.634277 | 9.441036 | 10.712816 | 8.513294 |
| **DM064** | 11.035885 | 10.770720 | 9.123161 | 10.884652 | 11.007795 | 9.842820 |
| **Mean** | **9.641650** | **9.752892** | **8.242630** | **10.273166** | **10.700489** | **8.904577** |
|  |  |  |  |  |  |  |
| **2 tailed p value** | **0.969166** | **0.385887** | **0.854082** | **0.869125** | **0.414625** | **0.444763** |

**Supplementary Table 5: Gene expression data for each gene in the MEK 6 gene score in the Docetaxel plus placebo group**. Shaded rows: patients with PR/CR to docetaxel plus placebo; unshaded: patients with PD at first assessment.

**Supplementary Figures**

**
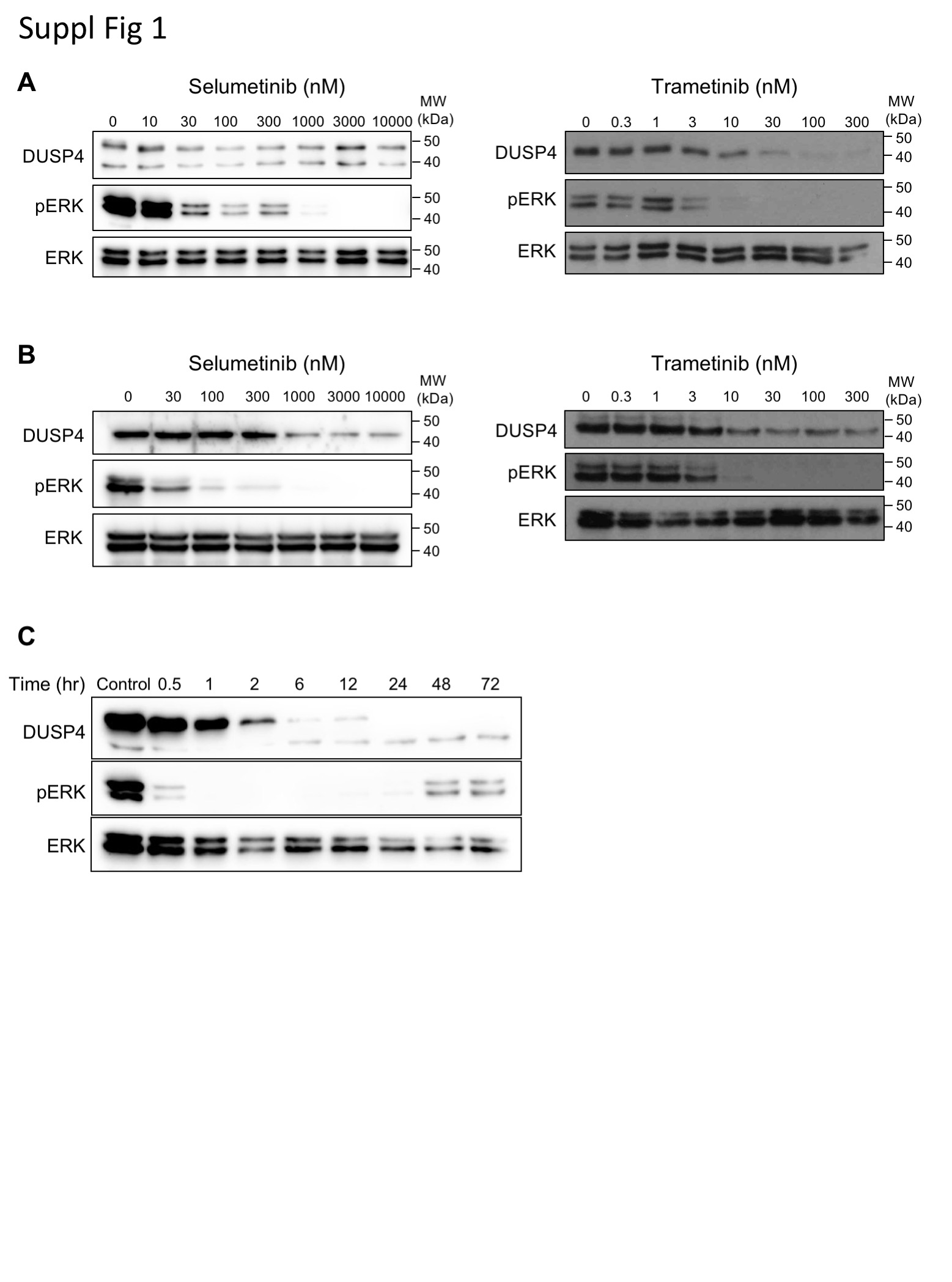
**

**Supplementary Figure 1. Concentration and time -dependent effect of MEK inhibition on ERK phosphorylation and DUSP4 expression in BRAF wild-type melanoma cells.** A, CHL-1 cells were treated with increasing concentrations of: left, selumetinib (n=1); right, trametinib (n=3, representative result is shown) for 1 hr before analysis by Western blot. B, SK-MEL-23 cells were treated with increasing concentrations of: left, selumetinib (n=1); right, trametinib (n=2, representative result is shown) and analysed as A. C, SK-MEL-23 cells were treated with 10nM trametinib for the indicated times. Control cells were treated with solvent and harvested at 24hr (n=1).


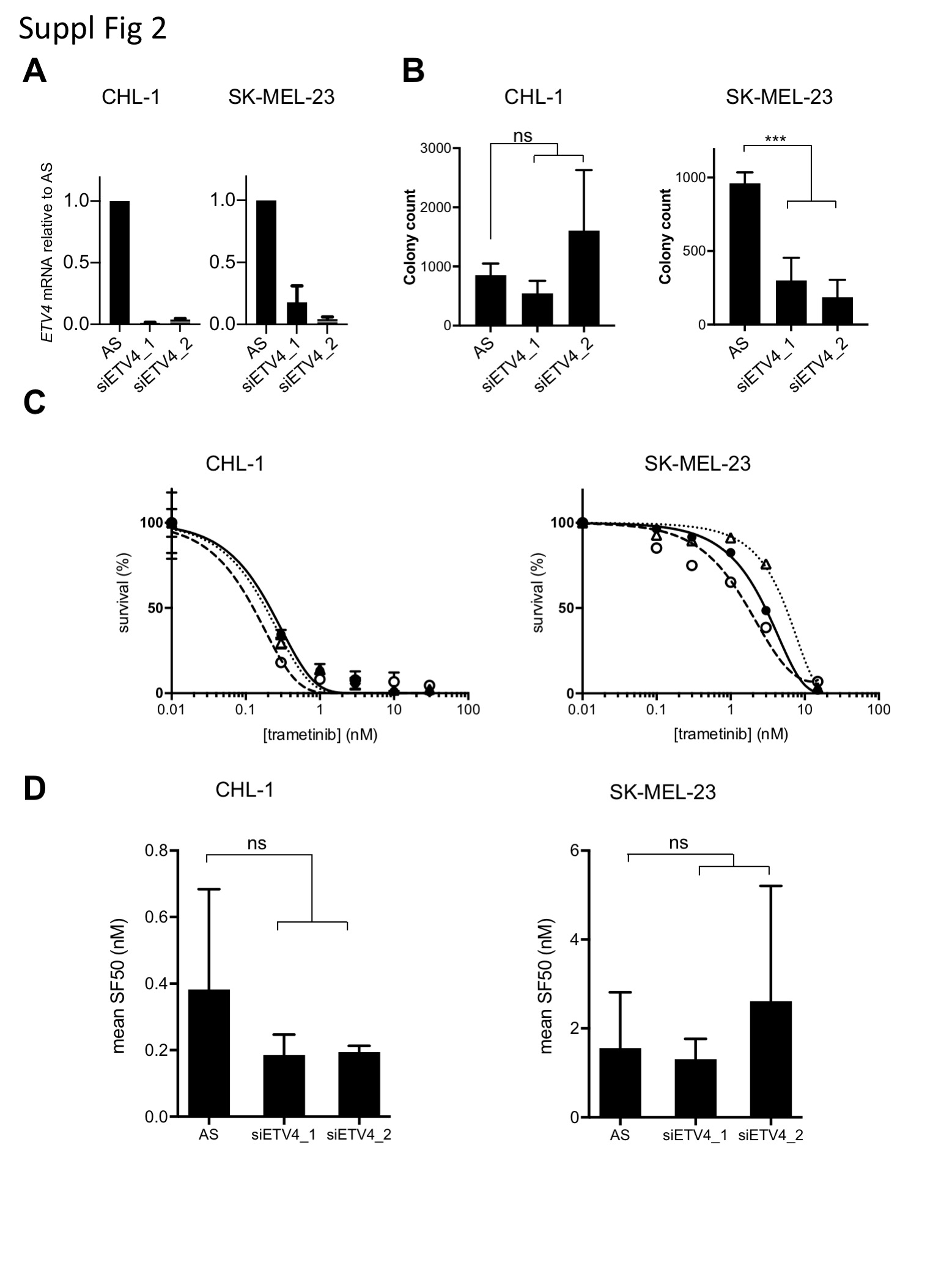
**Supplementary Figure 2: ETV4 depletion does not influence sensitivity to MEK inhibition in BRAF wild-type melanoma cells.** A, Quantification of *ETV4* expression determined by qRT-PCR in: left, CHL-1 and right, SK-MEL-23 cells transfected with control Allstars siRNA (AS) and 2 siRNAs against ETV4 (siETV4_1 and siETV4_2). Data are mean ± SEM from triplicate readings from 3 experiments. B, effect of ETV4 depletion by siETV4_1 and _2 on colony count for: left, CHL-1 and right, SKMEL-23 cells. In each case 3000 cells were originally seeded. C, compared with control Allstars siRNA transfectants (closed circles), ETV4 depletion using siETV4_1 (dashed line, open circles) and _2 (dotted line, open triangles) did not influence cell survival in response to trametinib in: left, CHL-1 and right, SK-MEL-23 cells. Values represent mean ± SD % survival in triplicate dishes for each condition. D, Summary of effect of ETV4 depletion on SF50 values for trametinib in: left, CHL-1 and right, SK-MEL-23 cells. Values represent mean ± SEM of triplicate values from three independent experiments for both cell lines.
